# Parametric Dynamic Distributed Containment Control of Continuous-Time Linear Multi-Agent Systems with Specified Convergence Speed

**DOI:** 10.3390/s23052696

**Published:** 2023-03-01

**Authors:** Fei Yan, Siyi Feng, Xiangbiao Liu, Tao Feng

**Affiliations:** College of Information Science and Technology, Southwest Jiaotong University, Chengdu 611756, China

**Keywords:** continuous-time MAS, containment control, dominant poles assignment, convergence speed

## Abstract

This paper focuses on the distributed containment control of continuous-time linear multi-agent systems (MASs) with multiple leaders over fixed topology. A parametric dynamic compensated distributed control protocol is proposed in which both the information from the observer in the virtual layer and actual adjacent agents are employed. The necessary and sufficient conditions of the distributed containment control are derived based on the standard linear quadratic regulator (LQR). On this basis, the dominant poles are configured by using the modified linear quadratic regulator (MLQR) optimal control and Geršgorin’s circle criterion, hence the containment control with specified convergence speed of the MAS is achieved. Another main advantage of the proposed design is, in the case of virtual layer failure, by adjusting parameters the dynamic control protocol reduces to static, and the convergence speed can still be specified through the dominant pole assignment method combined with inverse optimal control. Finally, typical numerical examples are presented to demonstrate the effectiveness of theoretical results.

## 1. Introduction

Since the past few decades, the distributed coordination of MASs has sparked a surge in interest from a wide variety of scientific fields for its possibilities of the extensive application seen in the Refs. [1,2,3,4]. As one of the most essential and fundamental problems in cooperative control of MASs, consensus control is to bring all agents into alignment on a feature or a state by designing appropriate distributed protocols [5], which has achieved a series of results [6,7,8,9,10]. Consensus studies mostly assume that there is no or only one leader in the MAS. However, in practical applications, MAS networks with multiple leaders are more typical. Then, the containment control arises, where the followers enter into a given geometric space spanned by the leaders.

There have been plenty of valuable outcomes. In the Ref. [11], a hybrid containment control algorithm was proposed to drive the followers into the convex hull spanned by the leaders. A second-order multi-agent containment control with random switching interconnection topology was considered in the Ref. [12]. In the Ref. [13], the robust containment problem with time-variant uncertainties was solved by an adaptive protocol. In the Ref. [14], the necessary and sufficient condition of containment control with time-delay was proved. In the Ref. [15], the fastest containment control of a discrete-time MAS was achieved under static protocol control, but the convergence speed of the system could not be adjusted arbitrarily. It can be seen that great efforts have been put into the system stability and static properties of MASs in containment control, while the adaptability and dynamic properties of the system have been little discussed.

In addition to the design of the distributed control protocol based on consensus, convergence speed is also an important indicator, describing how fast the agents reach an agreement, which is one of the most important research challenges in the design of distributed consensus algorithms for MASs. Many researchers in the Refs. [16,17,18,19,20,21] control the convergence speed by transforming or reconstructing topological structures since the network connectedness is critical in guaranteeing the convergence of consensus algorithms. However, this method is inapplicable for the MASs with fixed topology. According to the Ref. [3], the minimal non-zero eigenvalue of the Laplacian matrix can determine the convergence speed. It was figured out that in the Ref. [22] the convergence speed can be adjusted by configuring the closed-loop poles of the MAS. In the Ref. [23], the cooperative output regulation problem of linear MASs was solved by designing a distributed dynamic full information feedback control law with the distributed observer. Meanwhile, under the presented dynamic protocol, the idea of dynamic performance tuning by configuring poles has been proposed. However, the method in the Ref. [23] will not be able to achieve cooperative control of the system and adjustment of the dynamic performance, if the observer fails.

In this paper, we aim to propose a new distributed control protocol, which reduces the constraints of communication topology and provides better cooperative control performance. Moreover, the specified convergence speed of containment control will be achieved even if the distributed observer becomes invalid. The main contributions of the paper are reflected as follows:

(1) For a continuous-time linear MAS with multiple leaders over a directed topology, a new parametric dynamic compensated distributed control protocol for containment control is proposed. Compared with the containment control strategy in the Refs. [13,15], the co-states of agents in the virtual layer are introduced to reduce the limitation of the communication topology on the dynamic performance of the MAS. Compared with the protocol designed in the Ref. [23], the information about the actual state from the sensors of the physical layer is added, which can promote compatibility of the MAS and the adjustment to dynamic performance. Necessary and sufficient conditions for containment control are given based on the standard LQR design.

(2) Compared with the research of containment control in the Refs. [12,13,15], we focus on the arbitrary adjustment of the dynamic performance of the system. The accurate dominant pole configuration of the global closed-loop error system through the MLQR method and the Geršgorin’s circle criterion is used to achieve containment control with specified convergence speed. For the case where the virtual layer fails, the dynamic protocol will reduce to a static protocol based on the cooperative information from the physical sensors. Meanwhile, the convergence speed is specified by configuring the dominant poles of the resulting closed-loop error system combined with the inverse optimal regulator.

In the course of our research, we employed knowledge related to graph theory. A multi-agent system (MAS) can be abstracted in the form of a directed weighted graph G with *N* nodes $V=\left\{v_{1},v_{2},\cdots ,v_{n}\right\}$. The adjacency matrix is denoted by $A=\left[a_{ij}\right]\in R^{N\times N}$, if the information flows from node *j* to *i* then $a_{ij}>0$, otherwise, $a_{ij}=0$, $i,j\in N,N=\left\{1,2,\cdots ,N\right\}$. The set of neighbors of node *i* is denoted by $N_{i}$. Define the in-degree matrix as $D=diag\left\{d_{1},d_{2},\cdots ,d_{N}\right\},d_{i}=\sum_{j\in N_{i}}a_{ij}$ and the Laplacian matrix as L=D−A. The adjacency matrix A of an undirected graph must be symmetric, where $a_{ij}=a_{ji}$. When there exists a directed path from node *i* to every other node in the directed graph G, then G is said to have a spanning tree.

The remainder of the paper is organized as below. In Section 2, the main results will be proposed. Firstly, we introduce the parametric dynamic compensated distributed protocol and propose necessary and sufficient conditions for containment control over the directed graph. The specified convergence speed of the containment of agents is guaranteed by using the poles assignment technique for cases of the observers which are working and invalid. Section 3 gives three numerical examples to verify the developed theoretical results. Conclusions are given in Section 4.

*Notations:*$R^{m\times n}$ denotes the m×n real matrix space. $0_{m\times n}$ describes the zero matrix in $R^{m\times n}$. $I_{n}$ represents the *n* dimensional identity matrix in $R^{n\times n}$. $A^{T}$ denotes the transposition of matrix *A*, $A^{H}$ denotes the conjugate transposition of matrix *A*, and A>0 (A≥0) means matrix *A* is positive definite (semi-definite). $N_{i}$ denotes the *i*th node of the node set, $N_{D}$ denotes the leader nodes, and $N_{F}$ denotes the follower nodes; moreover, $N_{i}=N_{D}\cup N_{F}$. A matrix is Hurwitz if all of its eigenvalues have negative real parts.

## 2. Design of Dynamic Distributed Containment Control Protocol

### 2.1. Dynamic Containment Control

A continuous-time linear MAS with N+M nodes can be described by:

(1) $${\dot{x}}_{i}=Ax_{i}+Bu_{i}\;\forall i\in N,$$

where $x_{i}\in R^{n}$, $u_{i}\in R^{m}$, $N=\left\{1,2,\cdots ,N+M\right\}$ is node set, and the matrices *A*, *B* are the system matrix and control input matrix, respectively.

**Assumption** **1.** *The matrix pair (A,B) is controllable.*

Under Assumption 1, consider that there are *N* followers, which can be described by a directed graph while *M* leaders do not receive information from any other agent. Then the follower set and leader set are captured, which are, respectively, $F≜\left\{1,\cdots ,N\right\}$ and $D≜\left\{N+1,\cdots ,N+M\right\}$.

The dynamics of each leader and follower are:

(2) $${\dot{x}}_{i}=Ax_{i}\;\forall i\in D$$

(3) $${\dot{x}}_{j}=Ax_{j}+Bu_{j}\;\forall j\in F,$$

where $x_{i}$ is the state vector of leaders, $x_{j}$ is the state vector of followers, and $u_{j}$ is the control input vector of agent *j*.

The compact form of (2) and (3) can be written as:

(4) $$\dot{x_{l}}=\left(I_{M}⊗A\right)x_{l}$$

(5) $$\dot{x_{f}}=\left(I_{N}⊗A\right)x_{f}+\left(I_{N}⊗B\right)u,$$

where $x_{f}={\left(x_{1}^{T},x_{2}^{T},\cdots ,x_{N}^{T}\right)}^{T}$ is the global state vector of followers, $x_{l}=(x_{N+1}^{T},x_{N+2}^{T},\cdots ,$ $x_{N+M}^{T}{)}^{T}$ is the global state vector of leaders, and $u={\left(u_{1}^{T},u_{2}^{T},\cdots ,u_{N}^{T}\right)}^{T}$ is the global control input vector.

**Assumption** **2.** *For each follower in the MAS, there exists at least one leader that has a directed path to it.*

The communication topology graph of the MAS (1) is represented by G, and the structural characteristics of G can be described by a Laplacian matrix L. Since leaders are independent of each other, L can be written as a block matrix:

(6) $$L=\left[\begin{array}{cc} L_{f} & L_{l}\\ 0_{M\times N} & 0_{M\times M} \end{array}\right],$$

where $L_{f}\in R^{N\times N}$represents the information transmission situation related to followers, and $L_{l}\in R^{N\times M}$ represents the relation to leaders.

From (4), the following equation can be obtained by multiplying $\left(L_{f}^{-1}L_{l}\right)⊗I_{n}$ to both sides:

(7) $$\left[\left(L_{f}^{-1}L_{l}\right)⊗I_{n}\right]{\dot{x}}_{l}=\left(I_{M}⊗A\right)\left[\left(L_{f}^{-1}L_{l}\right)⊗I_{n}\right]x_{l}.$$

Under Assumption 2, all eigenvalues of $L_{f}$ have positive real parts, each entry of $-(L_{f}^{-1}L_{l})$ is nonnegative, and each row of $-(L_{f}^{-1}L_{l})$ has a sum of 1 [24], thus the linear combination of $x_{l}$ as follows can be referred to as the convex hull spanned by each element of $x_{l}$.

(8) $$-\left[\left(L_{f}^{-1}L_{l}\right)⊗I_{n}\right]x_{l}$$

**Lemma** **1** ([25])**.** *When $x_{f}\to -\left[\left(L_{f}^{-1}L_{l}\right)⊗I_{n}\right]x_{l}$, the states of all followers in the MAS will move into the convex hull spanned by leaders, hence the containment control is achieved.*

### 2.2. Parametric Dynamic Compensated Distributed Containment Control

Consider the following dynamic distributed control protocol with parameters:

(9) $$u_{i}=-cK\left[\omega_{i}\left(x_{i}-v_{i}\right)+\sum_{j\in N_{i}}a_{ij}\left(x_{i}-x_{j}\right)\right],\;i\in F,$$

where $v_{i}$ is the corresponding co-state for each follower which is generated by the following distributed dynamic compensator:

(10) $${\dot{v}}_{i}=Av_{i}-rW\left[\sum_{j\in N_{F}}a_{ij}\left(v_{i}-v_{j}\right)+\sum_{h\in N_{D}}a_{ih}\left(v_{i}-x_{h}\right)\right],$$

where the weight $\omega_{i}>0$, $K\in R^{m\times n}$, and $W\in R^{n\times n}$ are the feedback control gain matrices, the coupling coefficients c>0, r>0, $v_{i}$ is the corresponding co-state that the agent goes to track, $x_{h}$ represents the state of a particular leader node. The compact form of (9) and (10) can be written as:

(11) $$u=-c[\left(\Omega ⊗K\right)\left(x_{f}-v_{f}\right)+\left(L_{f}⊗K\right)x_{f}+\left(L_{l}⊗K\right)x_{l}]$$

(12) $${\dot{v}}_{f}=\left(I_{N}⊗A\right)v_{f}-r\left[\left(L_{f}⊗W\right)v_{f}+\left(L_{l}⊗W\right)x_{l}\right],$$

where

(13) $$\Omega =\mathrm{diag}\left\{\omega_{1},\omega_{2},\cdots ,\omega_{N}\right\}$$

and $v_{f}$ represents the co-state of each follower.

The proposed dynamic compensated distributed control law drives co-states into the convex hull, meanwhile each follower is able to follow the corresponding co-state, and thus dynamic containment control of the MAS can be achieved.

**Remark** **1.** *In the control protocol* (9)*, the first term implements followers tracking of the co-states; the second term achieves cooperative control by introducing actual relative information from physical sensors between agents. In a practical application scenario, such as a number of vehicles departing from different locations are required to drive into the safety zone formed by multiple mobile escort vehicles. At this time, the parameters of the cooperative control section can be regulated to achieve a special requirement of vehicles assembling into groups first and then driving into the safety zone, which increases the overall strike resistance of the convoy.*

Denote the error between the convex hull and co-states as δ, the error between each follower and the corresponding co-state as θ:

(14) $$\delta =v_{f}+\left(L_{f}^{-1}L_{l}⊗I_{n}\right)x_{l}$$

(15) $$\theta =x_{f}-v_{f}.$$

According to (7), the following equation holds:

$$\left(L_{f}^{-1}L_{l}⊗I_{n}\right){\dot{x}}_{l}=\left(L_{f}^{-1}L_{l}⊗A\right)x_{l},$$

then ${\dot{v}}_{f}$ can be written as:

(16) $${\dot{v}}_{f}=\left(I_{N}⊗A\right)v_{f}-r\left(L_{f}⊗W\right)\left[v_{f}+(L_{f}^{-1}L_{l}⊗I_{n})x_{l}\right].$$

Adding (7) and (16), we obtain:

$$\dot{\delta }={\dot{v}}_{f}+\left(L_{f}^{-1}L_{l}⊗I_{n}\right){\dot{x}}_{l}$$

(17) $$\dot{\delta }=\left[\left(I_{N}⊗A\right)-r\left(L_{f}⊗W\right)\right]\delta .$$

Similarly, $\dot{\theta }$ can be calculated as:

(18) $$\dot{\theta }={\dot{x}}_{f}-{\dot{v}}_{f}=\left(I_{N}⊗A\right)\theta -c[\left(\Omega ⊗BK\right)+\left(L_{f}⊗BK\right)]\theta -[c\left(L_{f}⊗BK\right)-r\left(L_{f}⊗W\right)]\delta .$$

Combining (17) and (18) yields the global closed-loop error system:

(19) $$\left[\begin{array}{c} \dot{\delta }\\ \dot{\theta } \end{array}\right]=\left[\begin{array}{cc} \Xi_{11} & 0\\ \Xi_{21} & \Xi_{22} \end{array}\right]\left[\begin{array}{c} \delta \\ \theta \end{array}\right],$$

where

$$\begin{array}{cc} \Xi_{11} & =\left(I_{N}⊗A\right)-r\left(L_{f}⊗W\right)\\ \Xi_{21} & =-c(L_{f}⊗BK)+r(L_{f}⊗W)\\ \Xi_{22} & =\left(I_{N}⊗A\right)-c\left(L_{f}+\Omega \right)⊗\left(BK\right). \end{array}$$

Denote the eigenvalues of $L_{f}$ and $\left(L_{f}+\Omega \right)$ as $\lambda_{i}$, $\chi_{i}$, respectively. Note that there exists nonsingular matrices Φ and Ψ such that:

(20) $${\left(\Phi ⊗I_{n}\right)}^{-1}\Xi_{11}\left(\Phi ⊗I_{n}\right)=\left[\begin{array}{cccc} A-r\lambda_{1}W\\ {}* & A-r\lambda_{2}W\\ \vdots & \vdots & \ddots \\ {}* & * & \cdots & A-r\lambda_{N}W \end{array}\right]$$

(21) $${\left(\Psi ⊗I_{n}\right)}^{-1}\Xi_{22}\left(\Psi ⊗I_{n}\right)=\left[\begin{array}{cccc} A-c\chi_{1}BK\\ {}* & A-c\chi_{2}BK\\ \vdots & \vdots & \ddots \\ {}* & * & \cdots & A-c\chi_{N}BK \end{array}\right].$$

Let $\tilde{\delta }={\left(\Phi ⊗I_{n}\right)}^{-1}\delta$, $\tilde{\theta }={\left(\Psi ⊗I_{n}\right)}^{-1}\theta$, then the error system (19) can be transformed into the following form:

(22) $$\left[\begin{array}{c} \dot{\tilde{\delta }}\\ \dot{\tilde{\theta }} \end{array}\right]=\left[\begin{array}{cc} {\hat{\Xi }}_{11} & 0\\ {\hat{\Xi }}_{21} & {\hat{\Xi }}_{22} \end{array}\right]\left[\begin{array}{c} \tilde{\delta }\\ \tilde{\theta } \end{array}\right],$$

where ${\hat{\Xi }}_{11}$, ${\hat{\Xi }}_{22}$ are shown as (20) and (21), respectively.

**Theorem** **1.** *Under Assumptions 1 and 2, the containment control can be achieved by the dynamic distributed protocol* (9) *if and only if the matrices*

$$\begin{array}{c} A-r\lambda_{i}W,\;i=1,\cdots ,N\\ A-c\chi_{j}BK,\;j=1,\cdots ,N \end{array}$$

*are Hurwitz.*

**Proof** **of Theorem 1.** The error system (19) is asymptotically stable, that is, (22) is stable, if and only if the following 2N matrices

$$\begin{array}{c} A-r\lambda_{1}W,\cdots ,A-r\lambda_{N}W,A-c\chi_{1}BK,\cdots ,A-c\chi_{N}BK \end{array}$$

are Hurwitz. Stability of the error system (19) and (22) indicates that ∥δ∥→0, ∥θ∥→0, which means the error between the convex hull and co-states δ, the error between followers and co-states θ tend to 0. It implies that containment control is achieved. □

In the following, we will prove the appropriate choice of the coupling gains *c* and *r*.

**Theorem** **2.** *Under Assumptions 1 and 2, using the control protocol* (9)*, where $W=R^{-1}P_{1}$ in which $P_{1}$ is a symmetric positive definite matrix and the solution of the following Riccati equation:*

(23) $$P_{1}A+A^{T}P_{1}-P_{1}R^{-1}P_{1}+Q=0.$$

*Similarly, $K=R^{-1}B^{T}P_{2}$ in which $P_{2}$ is the solution of $P_{2}A+A^{T}P_{2}-P_{2}BR^{-1}B^{T}P_{2}+Q=0$. If the coupling gains c and r satisfy:*

(24) $$r>\frac{1}{2\lambda_{\min}},\;c>\frac{1}{2\chi_{\min}},$$

*where λmin=minRe(λ1),Re(λ2),⋯,Re(λN) and χmin=min{Re(χ1),Re(χ2),⋯, Re(χN)}, then the global error system* (19) *is asymptotically stable, that is, the containment control is achieved.*

**Proof** **of Theorem 2.** According to Theorem 1, it is sufficient to make the subsystems $\left(A-r\lambda_{i}W\right)$ and $\left(A-c\chi_{i}BK\right)$ asymptotically stable. Take the subsystem $\left(A-r\lambda_{i}W\right)$ as an example, constructing the Lyapunov function:

$$V\left(x\right)={\tilde{\delta }}_{i}^{H}P_{1}{\tilde{\delta }}_{i}\;i=1,2,\cdots ,N,$$

where $P_{1}$ is the symmetric positive definite matrix and the solution of (23).

Taking the derivation of the function V(x) with respect to time yields:

(25) $$\dot{V}={\tilde{\delta }}_{i}^{H}P_{1}{\dot{\tilde{\delta }}}_{i}+{\dot{\tilde{\delta }}}_{i}^{H}P_{1}{\tilde{\delta }}_{i}={\tilde{\delta }}_{i}^{H}P_{1}\left(A-r\lambda_{i}W\right){\tilde{\delta }}_{i}+{\tilde{\delta }}_{i}^{H}{\left(A-r\lambda_{i}W\right)}^{H}P_{1}{\tilde{\delta }}_{i}={\tilde{\delta }}_{i}^{H}\left[P_{1}\left(A-r\lambda_{i}W\right)+{\left(A-r\lambda_{i}W\right)}^{H}P_{1}\right]{\tilde{\delta }}_{i}.$$

Replacing $R^{-1}P_{1}$ in (23) with the feedback gain matrix *W*, we have:

$$A^{T}P_{1}+P_{1}A=W^{T}RW-Q.$$

Similarly, the Riccati equation corresponding to subsystem $\left(A-r\lambda_{i}W\right)$ has the following form:

A−rλiWHP1+P1A−rλiW=−Q+1−2rRe(λi)WTRW.

Bringing into the equation (25), we can obtain:

(26) V˙=δ˜iH−Q+1−2rRe(λi)WTRWδ˜i.

From the Lyapunov theorem of asymptotic stability, if the subsystem $\left(A-r\lambda_{i}W\right)$ is to be asymptotically stable, then V(x) needs to satisfy $\dot{V}<0$. Due to *Q* being a positive definite matrix and *R* being a symmetric positive definite matrix, we can obtain that −Q<0 and $W^{T}RW\geq 0$.

To ensure $\dot{V}<0$, it is only needed to satisfy 1−2rRe(λi)<0. That is when the coupling gain *r* satisfies:

$$r>\frac{1}{2\lambda_{\min}},$$

where λmin=minRe(λ1),Re(λ2),⋯,Re(λN). Then the subsystem $\left(A-r\lambda_{i}W\right)$ is asymptotically stable with $W=R^{-1}P_{1}$.

Similarly, when the coupling gain *c* satisfies:

$$c>\frac{1}{2\chi_{\min}}.$$

The subsystem $\left(A-c\chi_{i}BK\right)$ is asymptotically stable with $K=R^{-1}B^{T}P_{2}$.

Therefore, when the global closed-loop error system is asymptotically stable, the MAS can achieve containment control. The proof is completed. □

### 2.3. Dynamic Distributed Containment Control with Specified Convergence Speed

It is known that the convergence speed is determined by the closed-loop poles that are closest to the imaginary axis. Therefore, the convergence speed will be specified by configuring the dominant poles of the global close-loop error system on dynamic containment control.

In the global closed-loop error system (22), the subsystems $\left(A-r\lambda_{i}W\right)$ and $\left(A-c\chi_{i}BK\right)$ are located in ${\hat{\Xi }}_{11}$ and ${\hat{\Xi }}_{22}$, respectively, so it is sufficient to design for these two blocks.

The eigenvalues of $\Xi_{11}$ (${\hat{\Xi }}_{11}$) without parameters $\omega_{i}$ are designed as non-dominated poles according to the MLQR [22] optimal control scheme.

Let $\bar{A}=A+\sigma I_{n}$, where there is the error system:

(27) $$\dot{\delta }=\left[\left(I_{N}⊗A\right)-r\left(L_{f}⊗W\right)\right]\delta ,$$

the subsystem is converted to

$$\bar{A}-r\lambda_{i}W=A+\sigma I_{n}-r\lambda_{i}W.$$

It is shown that when all eigenvalues lie to the left of the complex plane −σ, the error system (27) will be asymptotically stable and converge at a speed σ.

Therefore, if the value of σ is large enough, the poles of the virtual layer will move away from the imaginary axis and become non-dominant poles. At this time, the dominant poles of the closed-loop error system will be determined by $\Xi_{22}$, that is, subsystem $\left(A-c\chi_{i}BK\right)$.

For $\Xi_{22}$ (${\hat{\Xi }}_{22}$), the parameters $\omega_{i}$ are designed based on the Geršgorin circle theorem to configure the specified dominant poles.

**Lemma** **2.** *Under Assumption 2, for the appropriate choice of $\omega_{i}>0$, all eigenvalues of the matrix $\left(L_{f}+\Omega \right)$ are distinct and positive. Then, let $0<\chi_{1}<\chi_{2}<\cdots <\chi_{N}$, ∀μ>0, where the eigenvalues are all real and the ratio $\chi_{N}/\chi_{1}$ satisfies:*

(28) $$\frac{\chi_{N}}{\chi_{1}}<\frac{1+\mu }{1-\mu }.$$

**Figure 1:**
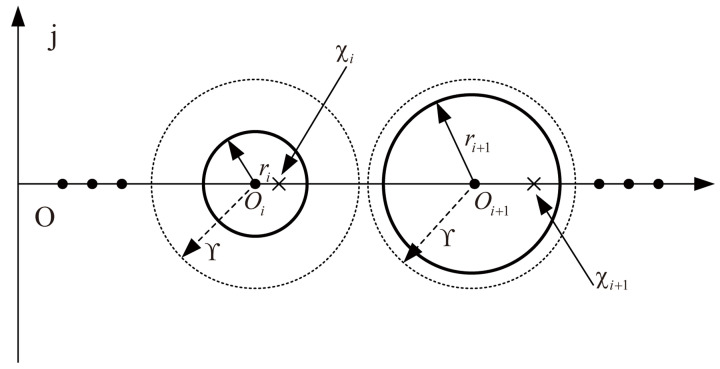
Gerschgorin circles.

**Proof** **of Lemma 2.** As shown in Figure 1, according to the Geršgorin circle theorem, the eigenvalue $\chi_{i}$ lies in the *i*th Geršgorin circle $Q_{i}=\left\{d|\left|d-O_{i}\right|\leq r_{i}\right\}$,where:

(29) $$O_{i}=\sum_{j\in N_{i}}a_{ij}+\omega_{i},\;r_{i}=\sum_{j\in N_{i}}a_{ij}\;i=1,2,\cdots ,N.$$

It is known that, all eigenvalues $\chi_{i}$ of $\left(L_{f}+\Omega \right)$ are distinct if all Geršgorin circles are separated, that is, $Q_{i}\cap Q_{j}=⌀,i\neq j$, which is equivalent to

$$\left|O_{i+1}-O_{i}\right|>\left|r_{i}+r_{r+1}\right|\;i=1,2,\cdots ,N.$$

For constants $\Upsilon >\max_{i\in N}\left\{r_{i}\right\}$ and any η>0, there always exists $\omega_{i}>0$ such that the following equation holds:

(30) $$O_{i}=\sum_{j\in N_{i}}a_{ij}+\omega_{i}=\eta +2i\Upsilon .$$

That is, $\omega_{i}$ satisfies the following equation:

(31) $$\omega_{i}=\eta +2i\Upsilon -\sum_{j\in N_{i}}a_{ij}.$$

Obviously, $\omega_{i}>0$. Using (30), then one has:

(32) $$\left|O_{i+1}-O_{i}\right|=2\Upsilon >\left|r_{i}+r_{r+1}\right|\;i=1,2,\cdots ,N,\;O_{1}=\eta +2\Upsilon >\Upsilon >0.$$

Thus all Geršgorin circles are separated. At this point, there must be at least one eigenvalue that lies into each Geršgorin circle; moreover, the first Geršgorin circle is located in the right-half of the complex plane. Therefore, all eigenvalues $\chi_{i}$ of $\left(L_{f}+\Omega \right)$ are distinct and positive. From (32), we can obtain:

$$0<\eta +\Upsilon <\chi_{1}<\eta +3\Upsilon$$

⋮

$$\eta +\left(2N-1\right)\Upsilon <\chi_{N}<\eta +\left(2N+1\right)\Upsilon ,$$

thus,

(33) $$\frac{\chi_{N}-\chi_{1}}{\chi_{N}+\chi_{1}}<\frac{N\Upsilon }{\eta +N\Upsilon }.$$

Denote that

$$\mu =\frac{\chi_{N}-\chi_{1}}{\chi_{N}+\chi_{1}},\;\eta_{0}=\frac{1-\mu }{\mu }N\Upsilon .$$

Then (33) holds when and only when $\eta >\eta_{0}$, that is, satisfies

$$\mu >\frac{\chi_{N}-\chi_{1}}{\chi_{N}+\chi_{1}}.$$

Simplify to obtain:

(34) $$\frac{\chi_{N}}{\chi_{1}}<\frac{1+\mu }{1-\mu }.$$

The proof is completed. □

**Remark** **2.** *For the readability of the readers, we here introduce the Geršgorin circle theorem for details. The Geršgorin circle theorem is used to bind the spectrum of a square matrix. Given a N×N matrix A with entries $a_{ij}$, for each i=1,⋯,N, we define*

$$\begin{array}{c} r_{i}=\sum_{j\neq i}\left|a_{ij}\right|,\;Q_{i}=\left\{d\in a_{ij}|\left|d-a_{ii}\right|\leq r_{i}\right\} \end{array}$$

*where $Q_{i}$ are denoted the Gerschgorin circles of A. Then every eigenvalue of A lies within at least one of the Gerschgorin circles $Q_{i}$.*

*We use the Geršgorin circle theorem in order to prove the following Theorem 3 and configure the dominant poles so that all the dominant poles can reach the specified position.*

**Theorem** **3.** *When the value of σ is chosen to be large enough and the poles of $\left(A-c\chi_{i}BK\right)$ are configured to the specified position, if the coupling gain c satisfies:*

(35) $$c=\frac{1}{\chi_{\min}},$$

*then the MAS can achieve dynamic containment control at a specified convergence speed.*

**Proof** **of Theorem 3.** According to Lemma 2 and its proof, the eigenvalues $\chi_{i}$ of $\left(L_{f}+\Omega \right)$ are nearly the same, so $1/\chi_{i}$ can be approximately equal to $1/\chi_{\min}$. If the coupling gain $c=1/\chi_{\min}$, the closed-loop dominant poles of $\left(A-c\chi_{i}BK\right)$ can be approximately equal to the eigenvalues of the matrix A−BK. Through the poles configuration, the absolute value of the real part of the conjugate eigenvalue of the matrix A−BK closest to the imaginary axis is ε. At this point, part of the poles of the closed-loop system (22) are located away from the imaginary axis to the left complex plane, and others converge to −ε, that is, the MAS (1) achieve containment control with the given convergence speed. The proof is completed. □

**Remark** **3.** *The MLQR [22] optimal control scheme can be used on poles’ configuration for the whole closed-loop error system* (22)*, and the convergence speed of the MAS can also be adjusted. However, for subsystem $\left(A-c\chi_{i}BK\right)$, a large overshoot occurs. Therefore the dominant pole configuration is applied to it by using Lemma 2.*

### 2.4. Regulation of the Convergence Speed in Case of Virtual Layer Failure

Consider a practical case where the observer fails in some agents, and where the information of co-states cannot be transmitted. At this time, the connectivity of the virtual layer topology is not guaranteed. However, the actual adjacent information in the physical layer can still be collected by sensors. Therefore, the control protocol can only use the adjacent information $x_{i}-x_{j}$ feedback to the system of the physical layer.

It can be regarded as the virtual layer subsystem being totally disabled. The dynamic distributed control protocol (9) reduces to a static form [26].

Under Assumption 1, let us investigate the containment control scheme and dynamic performance of the MAS (1) in Section 2.1. Without loss of generality, the matrices *A* and *B* are set as the following forms:

(36) $$A=\left[\begin{array}{cc} A_{11} & A_{12}\\ A_{21} & A_{22} \end{array}\right],\;B=\left[\begin{array}{c} 0\\ I_{m} \end{array}\right].$$

Similarly, *A*, *B*, and the control gain matrix $\tilde{K}$ satisfy the Riccati equation

(37) $$A^{T}P+PA-PBR^{-1}B^{T}P+Q=0.$$

Give a feedback control law

(38) $$\begin{array}{c} u_{i}=-\tilde{K}\sum_{j\in F\cup D}a_{ij}\left(x_{i}-x_{j}\right),\;\tilde{K}=\underline{\alpha }\left[\begin{array}{cc} F & I \end{array}\right] \end{array}$$

(39) $$\begin{array}{c} u=-\left(L_{f}⊗\tilde{K}\right)\left[x_{f}+\left({L_{f}}^{-1}L_{l}⊗I_{n}\right)x_{l}\right]. \end{array}$$

Similarly, with Section 2.2, the error between followers and convex hull spanned by leaders in MAS (1) is

(40) $$\zeta =x_{f}+\left(L_{f}^{-1}L_{l}⊗I_{n}\right)x_{l}.$$

(41) $$\dot{\zeta }=\left[\left(I_{N}⊗A\right)-\left(L_{f}⊗B\tilde{K}\right)\right]\zeta =\Xi \zeta .$$

Note that there exists a nonsingular matrix $\tilde{\Phi }$ such that:

(42) $${\left(\tilde{\Phi }⊗I_{n}\right)}^{-1}\Xi \left(\tilde{\Phi }⊗I_{n}\right)=\left[\begin{array}{cccc} A-\lambda_{1}B\tilde{K}\\ {}* & A-\lambda_{2}B\tilde{K}\\ \vdots & \vdots & \ddots \\ {}* & * & \cdots & A-\lambda_{N}B\tilde{K} \end{array}\right]$$

According to Theorem 1, the containment control can be achieved by the protocol (38) if and only if the matrices

(43) $$\begin{array}{c} A-\lambda_{i}B\tilde{K},\;i=1,\cdots ,N. \end{array}$$

are Hurwitz.

According to the algorithm in the Ref. [27], $\tilde{K}$ can be determined through the specified poles (or the desired transient characteristics). There exists a lower limit $\alpha_{0}$ for the value of $\underline{\alpha }$, that is, $\underline{\alpha }>\alpha_{0}$, while $\alpha_{0}$ is determined by the procedure in the Ref. [26]. It is known that from Section 2.2, the convergence speed relies on the locations of eigenvalues of $A-\lambda_{i}B\tilde{K},i=1,\cdots ,N$, whose asymptotic behavior shows in the following lemma.

**Lemma** **3** ([28])**.** Take the value of F in (38) such that the n−m eigenvalues of $A-B\tilde{K}$ are the specified closed-loop poles $\left\{d_{1}^{*},\cdots ,d_{n-m}^{*}\right\}$, then as $\underline{\alpha }\to \infty$:

(1) n−m eigenvalues of $A-\lambda_{i}B\tilde{K}$ satisfy $d_{i}\to d_{i}^{*},\;i=1,\cdots ,n-m$;

*(2) the rest m eigenvalues of $A-\lambda_{i}B\tilde{K}$ satisfy $d_{i}\to -\infty ,\;i=n-m+1,\cdots ,n$.*

Now the control protocol can be written as:

(44) $$u_{i}=-{\underline{\alpha }}_{i}\left[\begin{array}{cc} F_{i} & I \end{array}\right]\sum_{j\in F\cup D}a_{ij}\left(x_{i}-x_{j}\right),$$

whose compact form can be written as:

$$u=-\left(L_{f}⊗L_{\alpha }\left[F\;I\right]\right)\left\{x_{f}+\left(L_{f}^{-1}L_{l}⊗I_{n}\right)x_{l}\right\}.$$

**Theorem** **4.** *For a continuous-time MAS* (1) *with a given communication topology, under Assumptions 1 and 2, there exists a static control protocol* (44)*, such that the MAS (1) not only achieves containment control (2) but also achieves the specified convergence speed by configuring the n−m dominant poles to the desired locations.*

**Proof** **of Theorem 4.** For (1), according to Equations (38)–(43), it is similar to the proof of Theorem 1. For (2) [26], according to Lemma 3, under the condition $\underline{\alpha }\to \infty$, the eigenvalues $\left\{d_{1}^{*},\cdots ,d_{n-m}^{*}\right\}$ can be viewed as the dominant poles. By configuring the n−m closed-loop eigenvalues for each agent, the MAS can asymptotically achieve the desired performance (i.e., the specified convergence speed). □

Compared with the results of the research in the Ref. [23], according to Lemma 3, it is still possible to regulate the convergence speed of the MAS by configuring dominant poles based on the static control law (38), even the observer fails. However, compared with the dynamic protocol (9), this control method has disadvantages, such as complex structure and more severe overshoot in the initial phase of the response, seen in the simulation example of Section 3.3.

## 3. Simulation Examples

In this section, the correctness of the theoretical results and the effectiveness of the designed distributed control protocols will be verified by typical numerical examples.

**Figure 2:**
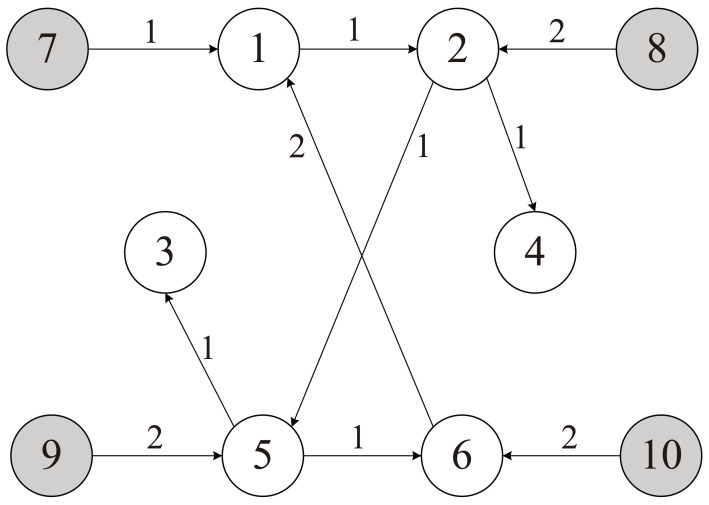
The communication topology of the MAS.

In a multi-vehicle escort application scenario, where the leaders are the escort vehicles and the followers are the protected vehicles, the whole convoy can be considered as a continuous-time MAS described by Figure 2 which has *M* leader agents (M=4) and *N* follower agents (N=6). The system matrix *A* and control input matrix *B* are set as follows:

$$A=\left[\begin{array}{cc} -0.01 & 0.02\\ 0.01 & -0.012 \end{array}\right],\;B=\left[\begin{array}{c} 0\\ 10 \end{array}\right].$$

The MAS can be written as:

$${\dot{x}}_{j}=Ax_{j}+Bu\;\forall j\in \left\{1,2,3,4,5,6\right\}$$

$${\dot{x}}_{i}=Ax_{i}\;\forall i\in \left\{7,8,9,10\right\}.$$

The communication graph G is given by Figure 2, then the Laplacian matrix L is given by:

$$L=\left[\begin{array}{cc} L_{f} & L_{l}\\ 0_{4\times 6} & 0_{4\times 4} \end{array}\right]$$

where

$$L_{f}=\left[\begin{array}{cccccc} 3 & 0 & 0 & 0 & 0 & -2\\ -1 & 3 & 0 & 0 & 0 & 0\\ 0 & 0 & 1 & 0 & -1 & 0\\ 0 & -1 & 0 & 1 & 0 & 0\\ 0 & -1 & 0 & 0 & 3 & 0\\ 0 & 0 & 0 & 0 & -1 & 3 \end{array}\right]$$

and

$$L_{l}=\left[\begin{array}{cccc} -1 & 0 & 0 & 0\\ 0 & -2 & 0 & 0\\ 0 & 0 & 0 & 0\\ 0 & 0 & 0 & 0\\ 0 & 0 & -2 & 0\\ 0 & 0 & 0 & -2 \end{array}\right].$$

Let $Q=I_{2}$, R=I.

Set the initial position of each vehicle as follows

$$\begin{array}{c} x_{j}=\left[\begin{array}{cc} 12 & -6\\ 6 & -20\\ 2 & 20\\ 8 & 4\\ -6 & -12\\ -8 & -8 \end{array}\right],\;x_{vj}=\left[\begin{array}{cc} 14 & -8\\ 8 & -38\\ 2 & 26\\ 10 & 6\\ -8 & -14\\ -10 & -10 \end{array}\right],\;x_{i}=\left[\begin{array}{cc} 2 & 4\\ -4 & 2\\ -1 & -10\\ 4 & -6 \end{array}\right] \end{array}$$

where $x_{j}$ and $x_{vj}$ are the initial positions of the followers, that is, the protected vehicles, in the physical and virtual layers, respectively, $x_{i}$ is the initial positions of the leaders, that is, the escort vehicles which form the convex hull.

### 3.1. Parametric Dynamic Compensated Distributed Containment Control

According to LQR optimal control, the feedback gain matrix of the physical and virtual layer can be calculated as:

$$K=\left[\begin{array}{cc} 0.6184 & 1.0000 \end{array}\right],\;W=\left[\begin{array}{cc} 0.9901 & 0.0148\\ 0.0148 & 0.9883 \end{array}\right].$$

Let $\Omega =I_{6}$, where it can be calculated that $\lambda_{\min}=1$, $\chi_{\min}=2$. According to Theorem 2, the coupling gains can be selected as c=15, r=0.6.

**Figure 3:**
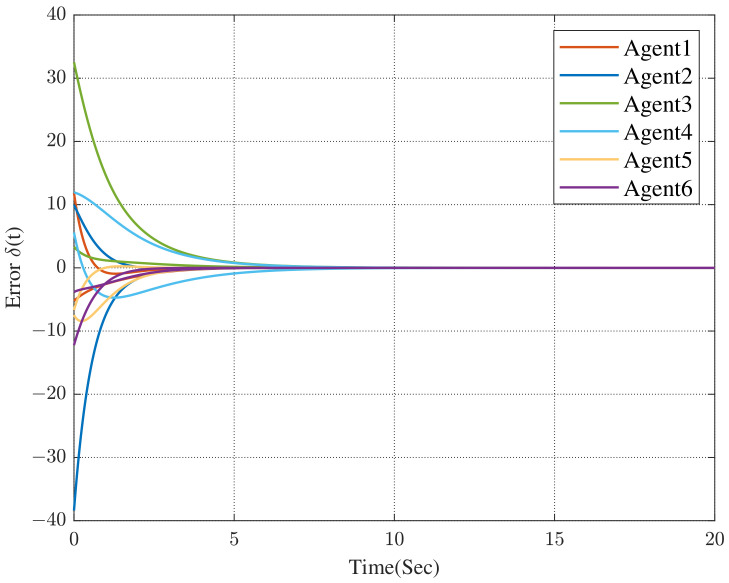
Error between co-states and the convex hull.

**Figure 4:**
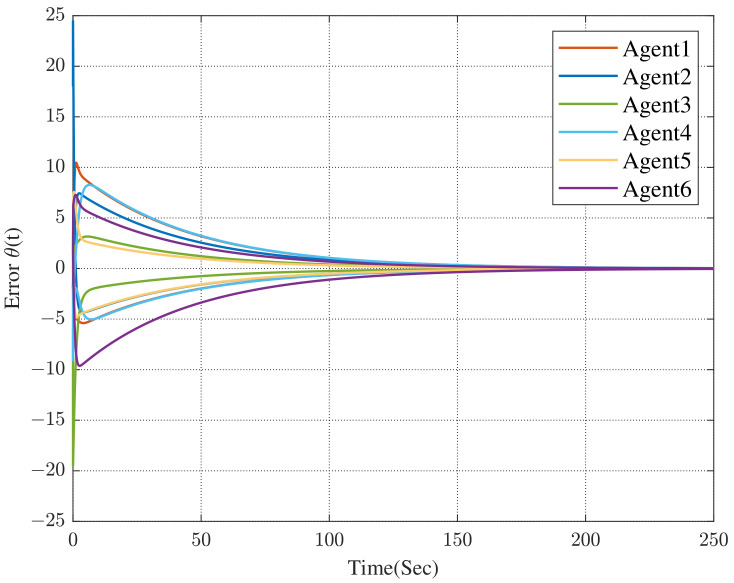
Error between followers and co-states.

Under the dynamic distributed control protocol (9), the convergence curve of the error system is respectively described in Figure 3 and Figure 4.

In Figure 3 and Figure 4, we can see that the co-states of the protected vehicles have entered into the convex hull formed by the escort vehicles, and the states of the protected vehicles have been the same as the co-states. It means that the protected vehicles have entered the convex hull, that is, the containment control has been achieved.

### 3.2. Containment Control with Specified Convergence Speed

Now we regulate the convergence speed of the MAS (1) by using the pole configuration method proposed in Section 2.3.

Let σ=10, then the poles of the global error system matrix (22) lie to the left of the complex plane −σ=−10.

Set the dominant poles as $\left\{-0.5+0.1j,-0.5-0.1j\right\}$, then the feedback gain matrix of the physical layer is calculated as K=[1.25150.0978].

According to Lemma 2, we set $\omega_{i}=10^{4}*\mathrm{diag}\left\{2,2.1,2.05,3.09,2.08,3\right\}$ then according to Theorem 3, the coupling gain $c=3.2361\times 10^{-5}$. At this point

$$c\chi_{i}\approx 1,\;A-c\chi_{i}BK\to A-BK,$$

which means that the poles of the physical layer subsystem are the configured poles, that is, the system converges at the specified speed.

**Figure 5:**
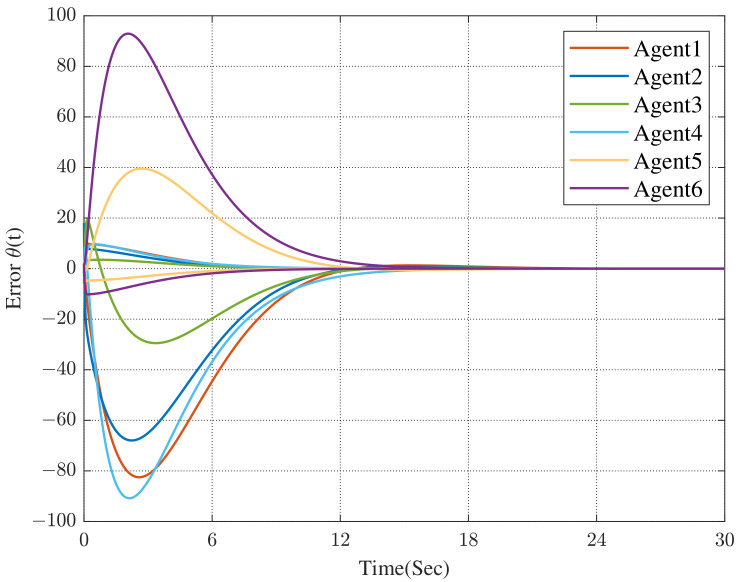
Convergence curves of the error between followers and co-states with specified convergence speed.

**Figure 6:**
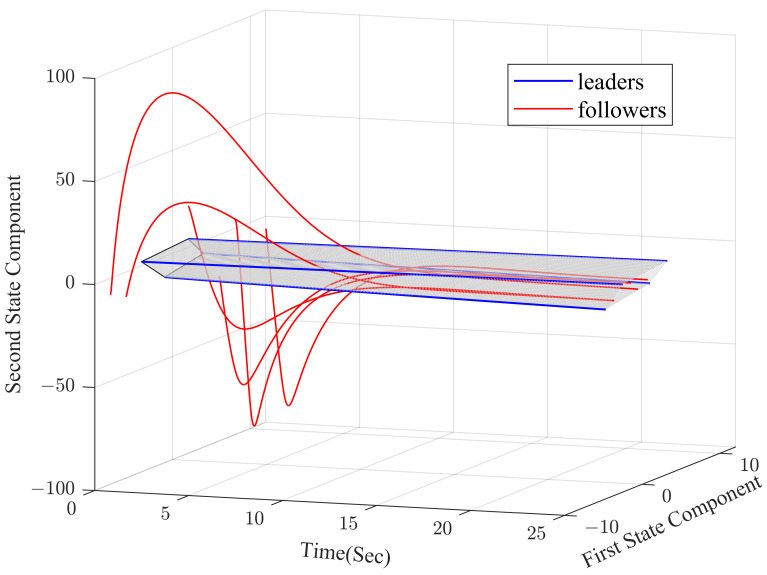
Agents converge into the convex hull (the two state components represent the position coordinates of each vehicle).

The convergence curve of the error system and the state transition curves of the protected vehicles are shown in Figure 5 and Figure 6, respectively.

Comparing Figure 4 with Figure 5, we can see that the convergence time has been significantly reduced (the former is approximately 230 s while the latter is approximately 24 s). In Figure 6, we can obtain that, all of the followers, that is, the protected vehicles are stabilized into the convex hull spanned by the leaders, that is, the escort vehicles under the control of the dynamic distributed control protocol (9) with a specified convergence speed.

### 3.3. Containment Control in Case of Virtual Layer Failure

Let

$$A=\left[\begin{array}{cc} -0.05 & 0.09\\ 0.1 & 0.08 \end{array}\right],\;B=\left[\begin{array}{c} 0\\ 1 \end{array}\right].$$

Set the dominant poles as −1, by taking sufficiently large value of $\underline{\alpha }$, the matrix $\tilde{K}$ is obtained $\tilde{K}=\left[52.7778\;5\right]$.

**Figure 7:**
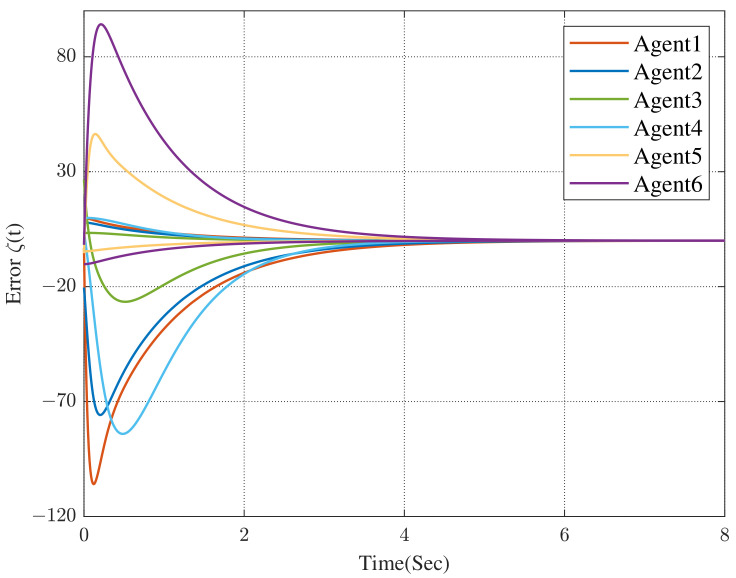
Error between followers and the convex hull.

**Figure 8:**
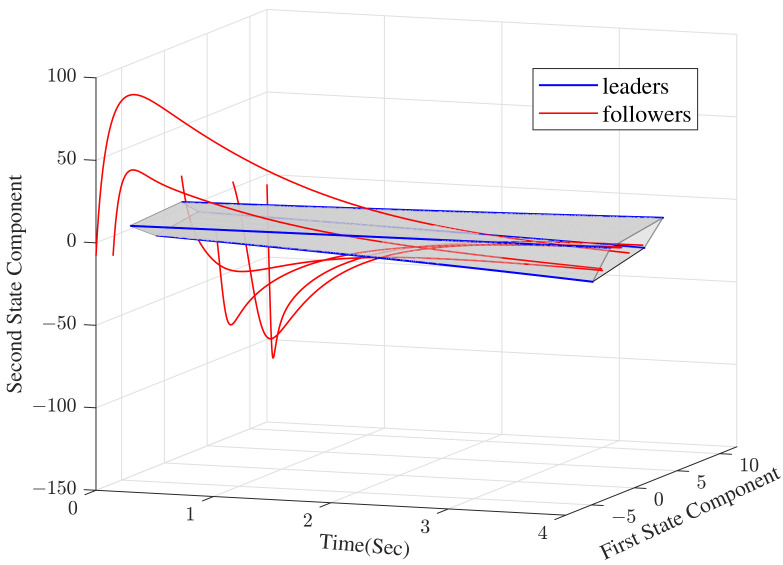
Followers converge into convex hull (the two state components represent the position coordinates of each vehicle).

The convergence curve of the new error system ζ and the state transition curve of the protected vehicles are shown in Figure 7 and Figure 8, respectively.

According to Figure 7 and Figure 8, apparently, when the observer fails, all the protected vehicles are still able to access the convex hull under the control of the static protocol (43) with a specified convergence speed.

## 4. Conclusions

This paper proposed a parametric dynamic compensated distributed control protocol with a co-state for each follower. For the containment control of the MAS, the necessary and sufficient conditions for taking values of the coupling gains have been derived. The dominant poles of the global closed-loop error system have been configured to specify the convergence speed of MAS. For the virtual layer subsystem, the poles have been configured as non-dominated poles by the MLQR optimal control; for the physical layer subsystem with parameters, the parameters have been designed based on the Geršgorin’s circle criterion to configure the desired dominated poles. When the virtual layer fails, the protocol reduced to a static control law to achieve containment control. Moreover, combined with inverse optimal control, the convergence speed can also be specified through dominant pole configuration. Simulation examples have been given to demonstrate the effectiveness of the developed design method.

## Data Availability

Not applicable.
